# Definition of the terms “acute” and “traumatic” in rotator cuff injuries: a systematic review and call for standardization in nomenclature

**DOI:** 10.1007/s00402-020-03656-4

**Published:** 2020-11-01

**Authors:** Jonas Pogorzelski, Bernd Erber, Alexander Themessl, Marco-Christopher Rupp, Matthias J. Feucht, Andreas B. Imhoff, Hannes Degenhardt, Markus Irger

**Affiliations:** 1 Department of Orthopedic Sports Medicine, Hospital Rechts Der Isar, Technical University of Munich, Ismaninger Street 22, 81675 Munich, Germany; 2 Department of Orthopaedics and Trauma Surgery, Medical Center, Faculty of Medicine, Albert-Ludwigs-University of Freiburg, Freiburg, Germany

**Keywords:** Rotator cuff tears, Acute, Traumatic, Acute on chronic lesions, Systematic review

## Abstract

**Background:**

Although of high relevance for clinical decision making, there exists no consensus throughout the literature of the terms “acute” and “traumatic” used in the classification of rotator cuff tears. With differing definitions, the comparability of outcome studies may be limited. The aim was to provide a detailed systematic review of the definitions used in the literature and present a suggestion for a standardization in nomenclature based on the findings.

**Methods:**

Four different internet databases were searched in February 2020 using the terms (“acute” OR “traumatic” OR “trauma” OR “athlete” OR “young”) AND (“rotator cuff tears” OR “rotator cuff tear” OR “rotator cuff” OR “rotator cuff rupture” OR “supraspinatus” OR “infraspinatus” OR “subscapularis” OR “teres minor”). Prospective, retrospective, cohort and case–control studies as well as case series were included. Systematic reviews, cadaveric or laboratory studies and studies on non-traumatic or non-acute rotator cuff tears were excluded.

**Results:**

The literature search conducted 10,349 articles of which 10,151 were excluded based on the title, 119 based on the abstract and 33 based on the manuscript. A total of 46 studies were finally included for review and subsequently analyzed. Overall, there exists no consensus neither on the term “acute” nor on “traumatic” in the context of rotator cuff tears in the literature. The time span for acute injuries ranged between 2 weeks and 6 months. For traumatic injuries, only 20% of the selected studies described a specific and adequate injury mechanism in combination with adequate imaging.

**Conclusion:**

The term “acute” should be reserved for RCT showing muscle edema, wavelike appearance of the central part of the torn tendon and joint effusion, which typically requires adequate imaging within 2 weeks from trauma. Repair of acute tears should occur within 8 weeks from trauma to benefit from possibly superior biological healing capacities. The term “traumatic” should be used for a sudden onset of symptoms in a previously asymptomatic patient, triggered by an adequate trauma, e.g., a fall on the retroverted arm with an axial cranioventral force or a traumatic shoulder dislocation.

## Introduction

Rotator cuff tears (RCTs) are a common shoulder pathology with at least 4.5 million physician visits only in the US every year [1]. Typically, rotator cuff tears occur more frequently in elderly than in younger patients because of an age-related degeneration of the rotator cuff tendons [2]. However, multiple reports observed a rising number of young patients below the age of 45 years suffering from RCT due to a traumatic incident [2–5] or chronic overuse, particularly in overhead athletes [6]. While the treatment of degenerative RCT in the elderly patient is controversial, there is consensus throughout the literature that surgery is generally advised for RCTs in young patients due to possible severe consequences, such as irreparable retraction of tendons, muscular atrophy and poor outcomes with the consequence of a rotator cuff arthropathy [6].

Although younger patients suffering from acute and traumatic RCT tend to have better tendon quality and better biological preconditions for tendon healing, the results published following rotator cuff repair in this population are heterogeneous and comparable to those of elderly patients [3, 7, 8]. A possible explanation might be that etiology is more critical for outcome than age. However, if there exists no uniform definition of the terms “acute” and “traumatic” throughout the literature, comparability of studies may be limited and proof of the aforementioned hypothesis hardly possible.

Therefore, the primary purpose of this study was to conduct a systematic review of the literature and summarize the available definitions of the terms “acute” and “traumatic” with respect to rotator cuff tears. The secondary purpose was to propose criteria for a more specific definition of both terms based on the gained insights. We hypothesized that there exists no consensus for both terms so far.

## Methods

### Inclusion and exclusion criteria

This systematic review was structured according to the PRISMA Checklist [9]. A literature search was performed focusing on studies investigating acute and traumatic rotator cuff tears. Prospective, retrospective, cohort and case–control studies as well as case series written in English and German were included. Systematic reviews, cadaveric or laboratory studies and studies on non-traumatic or non-acute rotator cuff tears were excluded.

### Search strategy

Two reviewers (*blinded for review*) independently searched electronic databases (PubMed, MEDLINE, COCHRANE Library, and EMBASE) on February 13th, 2020 using the following search term: (acute OR traumatic OR trauma OR athlete OR young) AND (rotator cuff tears OR rotator cuff tear OR rotator cuff OR rotator cuff rupture OR supraspinatus OR infraspinatus OR subscapularis OR teres minor). Furthermore, the references of the included full-text articles were searched for additional studies meeting the inclusion criteria.

### Study selection

Inclusion or exclusion of studies was based on the aforementioned criteria. Two reviewers (*blinded for review*) independently analysed titles and abstracts of all identified publications and the complete text of eligible articles. Divergences were resolved in collaboration with a third reviewer (*blinded for review*).

### Data extraction and quality assessment

The following data were abstracted from all included studies: study design, level of evidence, type of imaging used to detect the RCT, time frames used for definition of an acute injury and the definition of the term “traumatic” in the context of an RCT. Level of evidence was assigned according to the widely accepted common grading of Evidence Levels for Primary Research [10, 11]. The methodological quality of the included studies was assessed using the Methodological Index for Non-Randomized Studies (MINORS) [12]. The MINORS checklist consists of 12 items which are scored 0 (not reported), 1 (reported but inadequate) and 2 (reported and adequate) each with a maximum score of 16 for non-comparative studies (nc) and 24 for comparative studies (c). The methodological quality was categorized a priori as follows: a total score of 0–8 or 0–12 was considered as poor quality, 9–12 or 13–18 was considered as fair quality and 13–16 or 19–24 was considered as excellent quality, for non-comparative studies and comparative studies, respectively. Since this review aimed to analyze the definitions of the terms “acute” and “traumatic” for RCTs regardless of the patient outcome, MINORS could only evaluate the quality of the study but could not provide information on the quality of the definitions of the aforementioned terms.

## Results

**Fig. 1:**
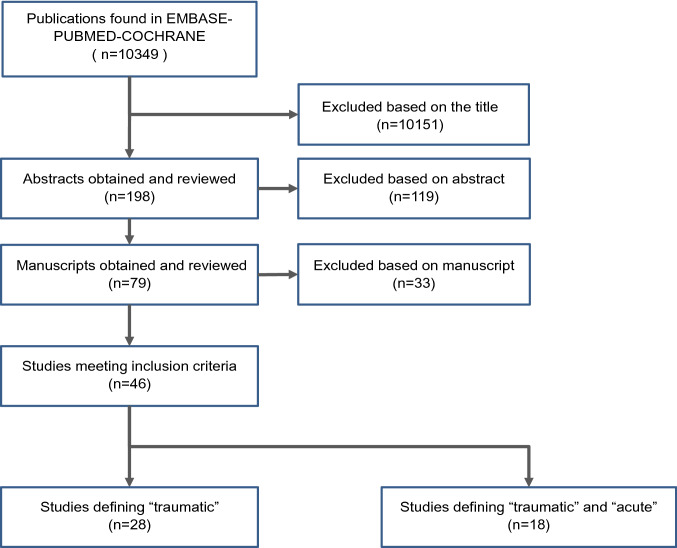
Flowchart of the study selection process

Overall, a total of 10,349 articles matched our search criteria on February 13th, 2020. After implementation of inclusion and exclusion criteria 10,151 were excluded based on the title, 119 based on the abstract and 33 based on the manuscript. As shown in Fig. 1, a total of 46 studies could be included with 28 studies defining only the term “traumatic” [3, 5, 13–38] and 18 studies defining both terms, “acute” and “traumatic” [39–56]. No study exclusively defining the term “acute” could be detected. The results of the included studies including MINORS score are presented in Table 1**.**

**Table 1:** Definitions of acute and traumatic RCT according to the reviewed literature

	References	Type of study (Level of evidence)	MINORS-Score	Number of patients	Imaging	Definition acute	Definition traumatic
1	Haviv et al. (2019) [35]	Retrospective case series (IV)	9 (nc)	38	MRI		“Rotator cuff tear following shoulder trauma in patient that was previously asymptomatic”
2	Bashir et al. (2019) [36]	Prospective cohort study (II)	12 (nc)	30	MRI		“Direct or indirect trauma to the shoulder.
3	Hasler et al. (2019) [37]	Retrospective case series (IV)	10 (nc)	19	MRI		“Clear traumatic event”
4	Plachel et al. (2019) [38]	Retrospective case series (IV)	12 (nc)	24	MRI		“A traumatic onset of symptoms was recorded in all patients (100%) with a high impact sport accident in 6 patients (30%) and with a low impact accident in 14 patients (70%). None of the patients reported on any shoulder complaints of the affected arm prior to injury”
5	Haviv et al. (2018) [25]	Prospective cohort study (II)	19 (c)	37	MRI		“A traumatic group included patients with sudden onset of symptoms that occurred after direct or indirect trauma to the shoulder without prior ongoing shoulder disability“
6	Aagaard et al. (2017) [13]	Prospective cohort study (II)	13 (nc)	79	MRI		“We defined acute rotator cuff tears as tears that occurred after direct or indirect trauma to the shoulder with sudden onset of symptoms in patients without ongoing shoulder discomfort or dysfunction“
7	Teratani (2017) [15]	Retrospective cohort study (III)	18 (c)	33	MRI		“In cases involving trauma at the onset of symptoms, the rotator cuff tear was determined to be traumatic and the mechanism of injury was recorded However, in this study, it was defined as traumatic rotator cuff tear only patients who were asymptomatic in the affected shoulder before the traumatic event and who were able to recall the specific date of the symptom“
8	Walcott et al. (2017) [16]	Retrospective case series (IV)	12 (nc)	7	MRI		“traumatic fall on abducted arm with a transtendinous rupture“
9	Tan et al. (2016) [14]	Retrospective case–control study (III)	19 (c)	811	Ultrasound		“We asked patients if they recalled a specific injury that caused their symptoms. If there was an injury, we recorded the date and mechanism of injury involved We categorized the mechanism of injury for patients with a history of trauma into 7 types (impacts to shoulders, sports injuries, assaults, falls, motor vehicle accidents, violent pulls, and unclear)“
10	Balke et al. (2016) [17]	Retrospective cohort study (III)	18 (c)	64	X-ray		“Patients were defined as traumatic cases if they reported a sudden injury with an accepted mechanism, e.g., a fall to the retroverted or externally rotated arm or a dislocation of the shoulder. Patients had to report pain and/or loss of function directly after trauma and to be previously without any complaints or impairments“
11	Loew et al. (2015) [31]	Prospective diagnostic study (II)	20 (c)	25	MRI		“Group A comprised 25 consecutive patients who underwent a shoulder injury with no history of problems with the involved joint. Trauma was defined as a sudden, unexpected external event determined by date and place. Only falls from standing height or greater onto the abducted outstretched arm were classified as trauma. Cases of simple contusions, a direct force on the shoulder (e.g., falling on the adducted arm), and distortion during active weight lifting were excluded“
12	Dilisio et al. (2015) [23]	Retrospective case series (IV)	12 (nc)	9	MRI		“Shoulder pain induced by a single traumatic episode; Patients younger than 25“
13	Kukkonen et al. (2013) [29]	Registry study (III)	18 (c)	112	X-ray		“In case of a clear trauma at the onset of symptoms, the rupture was regarded as traumatic and the mechanism was recorded“
14	Lin et al. (2013) [3]	Retrospective case series (IV)	10 (nc)	53			“Sudden traumatic etiology due to a fall, lifting heavy objects or an athletic event“
15	Bjornsson et al. (2011) [19]	Retrospective cohort study (III)	10 (nc)	42	MRI or Ultrasound		“Trauma to the shoulder, sudden onset of symptoms, asymptomatic shoulder before trauma, pseudoparalysis, full-thickness rotator cuff tear of at least 1 tendon with an acute appearance when sutured, and no signs off previous cuff tearing or other cuff pathology“
16	Bartl et al. (2011) [18]	Retrospective case series (IV)	12 (nc)	30	MRI and ultrasound		“Traumatic onset of the symptoms, mechanism described for every patient“
17	Safran et al. (2011) [33]	Retrospective case series (IV)	12 (nc)	51	Ultrasound		“Patients reported that a traumatic event initiated their symptoms in the year before their initial ultrasound examination“
18	Tambe et al. (2009) [34]	Retrospective case series (IV)	12 (nc)	11	MRI or Ultrasound		“All players sustained an injury during matchplay. Three players gave a convincing history of an Abduction-External rotation type impact without a dislocation, while one player sustained a true dislocation. Six players had sustained a direct impact injury, with their arm adducted and internally rotated at the time of impact.“
19	Krishnan et al. (2008) [5]	Retrospective case series (IV)	13 (nc)	22	MRI		“Patients recalled a single traumatic incident that incited their shoulder pain“
20	Ide et al. (2007) [27]	Retrospective case series (IV)	14 (nc)	20	MRI		“All patients had sustained an injury that was associated with the acute onset of shoulder pain and followed by functional impairment of the involved arm. Twelve patients were injured in a fall; in seven patients, the mechanism of injury was resistance to an external rotation force with the shoulder in a position of abduction and external rotation; and one patient was injured in a motor-vehicle accident“
21	Kreuz et al. (2005) [28]	Prospective cohort study (II)	14 (nc)	16	MRI and Ultrasound		“Isolated traumatic rupture. In 11 cases (including all the complete tears), the mechanism of injury was hyperextension or external rotation of the abducted arm. One patient injured his shoulder chopping wood, three were skiers and four were injured while attempting to prevent a fall down stairs. One athlete twisted his shoulder during a javelin throw and two handball players were blocked while throwing. Four patients with partial tears sustained a direct blow to the shoulder and one was involved in a motor vehicle accident.“
22	Braune et al. (2003) [21]	Retrospective cohort study (III)	20 (c)	20	MRI		“Those criteria included no pre-existing shoulder pain or malfunction, complete, sudden loss of shoulder function, sharp trauma- related pain with correlated dead arm sign in combination with an adequate trauma mechanism such as passive forced external or internal rotation with abducted or adducted arm, passive ventral, medial or caudal traction, axial compression in cranioventral or ventromedial direction, and the combination of traumatic shoulder dislocation Additional information was provided by the intraoperative macroscopic tear shape. References for a traumatic origin were the isolated complete tear of the subscapularis muscle and luxation of the head of the long biceps tendon with a subsequent rotator interval lesion. History of chronic shoulder pain and pre-existing shoulder malfunction, mechanical outlet impingement signs such as acromioclavicular joint arthritis or acromion traction osteophytes, patient age 50 years or older were used as exclusion criteria for a traumatic origin“
23	Goldberg et al. (2003) [24]	Retrospective case series (IV)	12 (nc)	6	MRI		“Four patients sustained the injuries while tackling, whereas two players fell with the ball in their arm, sustaining forced abduction injuries as they hit the ground. One patient sustained a frank dislocation at the time of the injury. All patients had also trauma before the index injury“
24	Hawkins et al. (1999) [26]	Retrospective case series (IV)	11 (nc)	16	X-ray		“Patients recalled an acute injury that heralded the onset of symptoms“
25	Deutsch et al. (1997) [22]	Retrospective case series (IV)	9 (nc)	13	MRI		“All patients sustained a traumatic injury before the onset of symptoms“
26	Payne et al. (1997) [32]	Retrospective cohort study (IV)	19 (c)	14			“Patients had acute onset of pain after a single traumatic event such as a direct blow or fall on the shoulder“
27	Le Huec et al. (1996) [30]	Retrospective case series (IV)	12 (nc)	10	MRI		“For each of the patients, the symptoms appeared after trauma: indirect trauma after a fall on the upper limb in abduction and internal rotation, fall with anterointernal dislocation, and fall with substantial torque in internal rotation with abduction“
28	Blevins et al. (1996) [20]	Retrospective case series (IV)	11 (nc)	10	MRI		“All patients recalled a specific event that precipitated pain and weakness in the injured shoulder. Each patient stated that he sustained a direct blow to the shoulder while playing football, either from a fall on to the shoulder or from direct contact with another player. One player had an anterior glenohumeral dislocation“
29	Raneboet al. (2019) [54]	Randomized controlled trial (I)	(rct)	58	MRI	< 3 months	“Patients without previous shoulder complaints seeking help for pain and/or decreased elevation after a shoulder trauma. Shoulder trauma was defined as any fall, impact, sudden pulling, or sudden stretching involving the symptomatic shoulder”
30	Aagaard et al. (2019) [52]	Prospective cohort study (II)	14 (nc)	62	MRI	< 6 weeks	“Patients who suffered from shoulder trauma, limited abduction and normal plain radiographs. Exclusion criteria include the following: chronic shoulder problems, rheumatoid arthritis, severe comorbidity, or previous surgery to the affected shoulder”
31	Aagaard et al. (2019) [53]	Prospective cohort study (II)	12 (nc)	32	MRI	< 6 weeks	“Patients who suffered from shoulder trauma, limited abduction and normal plain radiographs. Exclusion criteria include the following: chronic shoulder problems, rheumatoid arthritis, severe comorbidity, or previous surgery to the affected shoulder”
32	Spross et al. (2019) [56]	Retrospective case series (IV)	13 (nc)	21	MRI	< 2 months	“Acute trauma without previous shoulder pain, impairment, or surgery”
33	Artul and Habib (2017) [40]	Prospective cohort study (II)	14 (c)	112	Ultrasound	< 2 months	“An acute traumatic RCT was defined as when the clinical history revealed a distinct injury in a previously asymptomatic shoulder“
34	Abechain et al. (2017) [39]	Retrospective cohort study (III)	17 (c)	35	MRI	< 6 months	“The traumatic rotator cuff tear group was defined by trauma followed by acute shoulder pain associated with impaired active range of motion of the affected limb. This trauma can be a cause of a medial rotation or lateral force with the arm adducted or abducted, a ventral, medial or caudal passive draw force, an axial compressive force toward the cranial and ventral or ventromedial direction or secondary to a shoulder dislocation. It was expected that the patient did not have any pain before the trauma“
35	Duncan et al. (2015) [44]	Prospective cohort study (II)	19 (c)	20	MRI and Ultrasound	< 6 months	“This was defined as a sudden episode of shoulder pain precipitated by a traumatic episode resulting in a deterioration in shoulder function with self-reported normal shoulder function before injury“
36	Loew et al. (2014) [48]	Prospective case control study (II)	21 (c)	25	MRI	< 6 weeks	“First appearance of shoulder disability caused by a fall or violence on the joint“
37	Butler et al. (2013) [43]	Retrospective case series (IV)	12 (nc)	15	MRI	< 6 weeks	“Self-reported injury, no previous shoulder pain“
38	Petersen et al. (2011) [55]	Retrospective case control study (IV)	16 (c)	36	MRI	< 4 months	“All patients in this study had a previously asymptomatic shoulder prior to injury and had sustained an acute, traumatic, full-thickness rotator cuff tear that resulted in immediate pain and the inability to achieve greater than 90 of active abduction of the shoulder. In all instances, the patients experienced a traumatic injury from a variety of mechanisms that resulted in their shoulder symptoms. All study patients had an asymptomatic, fully functional shoulder prior to injury”
39	Hantes et al. (2011) [46]	Prospective cohort study (II)	19 (c)	35	MRI	< 3 weeks	“Diagnosis of a traumatic RCT was based on the recommendations of the German Association of Shoulder and Elbow Surgery. These criteria included no pre-existing shoulder pain or malfunction, complete, sudden loss of shoulder function, sharp trauma-related pain with correlated dead arm sign in combination with an adequate trauma mechanism such as passive forced external or internal rotation with abducted or adducted arm, passive ventral, medial or caudal traction, axial compression in cranioventral or ventromedial direction, and the combination of traumatic shoulder dislocation. In the contrary, history of chronic shoulder pain or pain with onset irrelevant to the traumatic incident, pre-existing shoulder malfunction, acromioclavicular joint arthritis, and acromion osteophytes were criteria of non-traumatic RC tear“
40	Sorensen et al. (2007) [49]	Prospective diagnostic study (II)	12 (nc)	109	Ultrasound	< 2 weeks	“Patients were included if they had had an acute shoulder trauma to a previous healthy shoulder“
41	Lähteenmäki et al. (2006) [47]	Retrospective case series (IV)	11 (nc)	29	Arthro-graphy, ultrasound or MRI	< 3 weeks	“The patients had a history of trauma with the acute onset of symptoms accompanied by a full-thickness tear of the rotator cuff“
42	Braune et al. (2000) [42]	Prospective cohort study (II)	18 (c)	12	MRI	< 12 weeks	“An adequate trauma with an defined and energetic appropriate accident caused a sudden and durable pain and loss of function; no pain before trauma“
43	Teefey et al. (2000) [50]	Retrospective cohort study (III)	12 (nc)	24	Ultrasound	< 6 months	“An acute RCT was considered to be present when (1) the clinical history revealed a distinct injury within 6 months from the time of operation in a previously asymptomatic shoulder and (2) the operative findings showed blunt, frayed cuff edges, tendon quality and thickness comparable to those of an intact cuff, and a freely mobile cuff“
44	Zanetti et al. (1999) [51]	Prospective cohort study (II)	20 (c)	24	MRI	< 6 weeks	“Rotator cuff tear by a recent (< 6 weeks) trauma, clinically normal shoulder before trauma (absence of prior injury, shoulder pain, functional abnormality, or surgery)“
45	Farin and Jaroma (1995) [45]	Prospective diagnostic study (II)	13 (nc)	94	Ultrasound	< 3 weeks	“Patients experienced acute trauma“
46	Bassett and Cofield (1983) [41]	Prospective cohort study (II)	10 (nc)	37		< 3 weeks	“Patients had a significant trauma“

For the diagnostic assessment of the rotator cuff, 31 studies within the included publications (67%) used magnetic resonance imaging (MRI) [5, 13, 15, 16, 18, 20–25, 27, 28, 30, 31, 35–39, 42–44, 46, 48, 51–56]. In an additional six studies (13%), ultrasound was used for detecting a torn rotator cuff [14, 33, 40, 45, 49, 50]. Three studies (7%) reported that either MRI or ultrasound was adequate for evaluation of an RCT [19, 34, 47]. Another three studies (7%) relied on X-rays only [17, 26, 29]. A total of three studies (7%) did not report use of imaging modalities at all [3, 32, 41].

Of the studies included in this review, histological examinations were only performed in a single study [42]. In this study, two samples of the tendons of each patient were taken and sent to different laboratories for evaluation to distinguish between acute or chronic RCTs based on the absence or presence of hemosiderin. Due to the heterogeneous and contradictory results in this study, the authors later decided to exclude histopathological examinations in their study [42].

### Results for defining the term “acute”

According to the 18 articles defining the term “acute” with regard to RCTs, the time range was defined between less than 2 weeks up to 6 months (Table 1) [39–56]. These studies can be further subdivided into ten studies, using a period of a minimum of two up to a maximum of 6 weeks as time span for an acute RCT [41, 43, 45–49, 51–53] and eight studies determining a time span from 2 to 6 months for their definition of “acute” [21, 39, 40, 44, 50, 54–56].

Ten studies contained specific explanations of their definition of the term “acute” [41, 43–47, 52, 53, 55, 56]. Studies performed by Basset and Cofield [41] and Farin and Jaroma [45] found lower degrees of retraction of the tendon and less scarring of the joint within the first 3 weeks after trauma and, therefore, proposed that surgical repair would be easier in this time frame. Therefore, the authors suggest, that 3 weeks should be considered as the time span for the definition of an acute RCT [41, 45]. Two studies further recommended surgery in a period of less than 3 weeks, referring to the study of Basset and Cofield [41, 46, 47]. Three articles set their definition of an acute RCT within 6–8 weeks after injury, using the average time span from several other clinical studies as a rationale for their definition [43, 53, 56]. Duncan et al. [44] argued that the evidence for defining acute and chronic remained unclear, using 6 months as a distinctive threshold. One study referred to the current Swedish national guideline that recommends surgery within 6 weeks [52]. Petersen et al. [55] reported favorable results for patients with RCTs treated within 4 months. The remaining eight studies provided no concise explanation for their definition of an acute RCT [39, 40, 42, 48–51, 54].

### Results for defining the term “traumatic”

The term “traumatic” was defined in all 46 included studies [3, 5, 13–56]. The definitions of the term “traumatic” used in each publication are shown in Table 1**.**

For the definition of a traumatic lesion of the rotator cuff, all included studies required a sudden onset of symptoms following a patient-reported trauma to the shoulder [3, 5, 13–56].

Furthermore, in 24 studies (52%) only RCTs in previously asymptomatic patients were considered traumatic lesions [13, 15, 17, 19, 21, 25, 31, 35, 38–40, 42–44, 46, 48–56].

The trauma mechanism of the injury was detailed in 23 studies (50%) [3, 13–18, 20, 21, 24, 27–32, 34, 38, 39, 46, 48, 54, 55]. Of these, eleven publications (24%) classified injuries with a specific and adequate injury mechanism as traumatic [3, 13, 16, 17, 21, 30, 31, 34, 39, 46, 54], whereas twelve studies (26%) described the trauma mechanism without assessing the respective adequacy for the definition “traumatic” [14, 15, 18, 20, 24, 27–29, 32, 38, 48, 55]. More extended criteria were used in 14 publications (30%) [3, 16, 19, 21, 23, 39, 42, 44, 46, 47, 50, 52, 53, 55]. One study investigating traumatic tears only included patients younger than 25 years [23]. Loss of function was required in nine studies [19, 21, 39, 42, 44, 46, 52, 53, 55]. The intraoperative macroscopic or radiologic tear morphology was specified in five studies [3, 16, 21, 47, 50]. Furthermore, four publications defined RCTs as non-traumatic, if the patients had intraoperative or radiologic signs of previous shoulder dysfunction [19, 21, 46, 50].

## Discussion

The main finding of this review is, that there exists no consensus on neither the term “acute” nor “traumatic” in the context of rotator cuff tears in the literature. In the studies reviewed for this article, RCT was defined as an acute injury with a time span of less than 2 weeks up to 6 months [39–56]. For defining a traumatic RCT, only 20% of the selected studies included both adequate injury patterns and appropriate radiological diagnostic modalities [13, 16, 21, 30, 31, 34, 39, 46, 54].

### Definition of the term “acute”

Similar to the biologic repair process of any other tendon tissue, the healing process of rotator cuff tears is time-dependent and commonly divided into three consecutive phases: (1) inflammation, (2) repair/proliferation and (3) remodeling [57–60]. Each of the phases depends on the interplay of different cell populations, cytokines and growth factors [61]. It is important to note, that the expression of growth factors and cytokines is highly time-dependent.

First of all, immediately after an injury, bleeding leads to the accumulation of cytokines and growth factors such as fibrin, fibronectin, interleukin1-ß (IL1-ß), IL-6, tumor necrosis factor α (TNF-α), insulin-like growth factor-1 (IGF-1), platelet-derived growth factor (PDGF), basic fibroblast growth factor (bFGF) and transforming growth factor ß (TGF)-β. These growth factors peak in expression after 7 days [62, 63] and activate fibroblasts, which play a key role in the activation of the healing process, angiogenesis and remodeling of the delevoping extra-cellular matrix [61, 64, 65]. Furthermore, the release of these proteins results in the attraction of inflammatory cells such as neutrophils, macrophages and platelets, initiating the inflammatory phase, which lasts for approximately 7 days [57–59, 66].

This initial inflammatory phase is followed by a phase of repair and proliferation, lasting for approximately 2 weeks after the injury [57–60]. Herein, cell proliferation and angiogenesis are regulated by vasoactive and chemotactic factors such as VEGF and TGF-β, which activate collagen synthesis, angiogenesis and termination of cell proliferation. All of those factors peak 10 days after injury [62, 63]. In this phase, fibroblasts begin to produce collagen, mainly type I and III [57, 58, 62, 63, 67, 68]. While collagen type I is predominant in healthy tendons, the developing scar tissue contains a higher proportion of collagen type III [66].

The subsequent third phase of remodeling begins after a few weeks and can continue for several years [36, 66]. As a part of the remodeling phase, IGF-1 release is predominant 3 weeks after injury and further stimulates the collagen synthesis [58]. As part of this process, a shift to a more physiologic distribution between collagen types I and III is occuring, resulting in an increasingly organized extra-cellular matrix [66, 67]. However, after a tear at the tendon-bone interface, the biological processes are not able to restore the native composition of the structure, resulting in a fibrovascular scar [69]. For example, several studies in animal models have shown that the regenerative tissue formed in supraspinatus tendons in rats is of inferior mechanical and histological quality compared to its native composition [60, 67]. A decrease of all measured growth factors to control-group level was observed after 16 weeks, which could be considered as the biological equivalent to the completion of the healing process [63].

Radiologic imaging is helpful to detect RCTs and to distinguish between acute and chronic tears. Based on the literature, the MRI is considered the standard imaging technique to reliably diagnose an RCT [70–72]. As MRI is expensive, time-consuming and not ubiquitously available, ultrasound performed by experienced musculoskeletal clinicians is considered a valid imaging tool with reliable results for detecting RCT [73–75]. In case of an acute and traumatic rotator cuff tear, muscle edema and joint effusion and a wavelike appearance of the central part of the torn tendon, which is also known as the “kinking sign”, can be detected [31]. In contrast, muscular atrophy and fatty infiltration are safe signs for a chronic component of the RCT, which can be visualized considerably more precise by MRI. However, the degree of tendon retraction and thinning of the tendon are unspecific signs and insufficient to distinguish between acute and chronic tears [31]. In the included studies, either ultrasound or MRI was used in 40 of 46 studies (87%) [5, 13–16, 18–25, 27, 28, 30, 31, 33–40, 42–56]. In the remaining six studies (13%), only X-ray imaging was used or no imaging modality was employed at all [3, 17, 26, 29, 32, 41].

Apart from radiologic imaging, histological investigations may provide information regarding the causality of the RCT. For example, thinning and disorientation of collagen fibers, vascular proliferation, calcification, myxoid degeneration and pronounced chondroid metaplasia can be considered as signs of pre-existing damage or degenerative changes of the rotator cuff tendons [76]. Acute injuries on the other hand, are characterized by hemosiderin appearence, hypervascularization and regenerative changes [77, 78]. While histopathological examinations may facilitate an ex post classification of the RCT type, it is not feasible to exclusively rely on histopathological examinations, when differentiating between acute and chronic RCTs—due to the heterogeneous and contradictory results and general impracticality in the clinical setting [42].

### Definition of the term “traumatic”

Several factors, including history of trauma and mechanism of injury, contribute to the definition of a traumatic RCT. In terms of injury mechanisms, it is important to define truly traumatic tears more precisely and to distinguish them from RCTs resulting from inadequate injury mechanisms such as contusions, minor impacts or active muscle contractions during weight lifting [79, 80].

From a biomechanical point of view, various injury patterns can lead to excessive eccentric loading and overstretching of the tendons of the rotator cuff resulting in a traumatic RCT [80]. These mechanisms include shearing of the tendons on the glenoid rim, when the maximal tolerated rotation angle is exceeded. For example, a staircase fall with the attempt to prevent falling by holding on the railing results in a passively forced external or internal rotation and abduction with a massive overstretching of anterocranial or posterocranial structures, respectively, possibly leading to a RCT [79, 80]. In addition, axial compression and passive traction can lead to an RCT—a mechanism present in incidents such as a fall on the retroverted arm, a high velocity accident or unprepared catching of a heavy load. Similarly, a shoulder dislocation represents an adequate mechanism capable of causing injury to a healthy rotator cuff tendon [21, 31, 39, 46, 80].

While all included studies reported shoulder trauma as the cause of injury [3, 5, 13–56], only 24 of the 46 studies (52%) explicitly reported, that the injuries occurred in previously asymptomatic patients [13, 15, 17, 19, 21, 25, 31, 35, 38–40, 42–44, 46, 48–56]. In addition, only eleven studies (24%) described a specific injury mechanism, that could be considered adequate for a traumatic RCT [1, 4, 9, 16, 33, 48, 50, 52, 66, 71, 75].

### Call for standardization in nomenclature

After reviewing the existing literature, we believe that the terms “acute” and “traumatic” must be considered in combination. The underlying rationale is to exclude so called “acute on chronic”-lesions, where an inadequate trauma leads to a rupture of a pre-damaged rotator cuff tendon [31]. Those tears may not have the same healing capacities as primarily traumatic RCTs. To categorise an RCT as “traumatic”, the patient-reported trauma needs be adequate and follow a biomechanically plausible injury mechanism. There must be a clear differentiation from minor traumatic incidents or inadequate injury mechanisms, indicative of a non-traumatic tear of other etiology. Moreover, for an RCT to be considered traumatic, a previously asymptomatic patient needs to report a sudden onset of symptoms in the shoulder after the trauma.

To be able to detect a truly traumatic and acute RCT, imaging should be performed within the first 2 weeks after injury, as the aforementioned radiologic properties of acute RCT such as muscle edema, joint effusion and the kinking sign can be visualised best in this time interval. In general, RCT should be visualised by ultrasound or MRI for an adequate statement about the ruptured tendon and possible concomitant pathologies. MRI is considered the gold standard imaging modality for RCT, as it allows further assessment of intraarticular structures and, more importantly, enables to detect signs of degenerative changes [70–72]. Histological investigations do not seem to be mandatory, as no clear evidence could be produced, that this modality is a reliable and practial tool to distinguish between acute and chronic rotator cuff tears.

Nonetheless, it must be taken into account, that due to the very complex interaction of growth factors, cytokines and involved cells, no comprehensive statement about the healing mechanisms and the ideal therapeutic time span for surgery in acute RCT can be made. Yet taking the decrease of most growth factors, cytokines and the collagen shift within the tear zone 8 weeks after trauma into account [58, 59, 63], it must be assumed that the healing capacity is significantly inferior after this period. Therefore, we recommend a time span of no more than 8 weeks to attempt a surgical repair of an acute RCT, to still benefit from superior biological healing capacities posttraumatically. A time period of more than 4 months after trauma appears to be generally unfavorable, due to the dynamics of the aforementioned physiological healing capacities [21, 39, 40, 44, 50, 54–56].

Our proposed definitions may help standardising the use of the terms “acute” and “traumatic” and, therefore, improve future understanding of the outcomes following rotator cuff repair, but further specification and elucidation of the exact healing processes is required. We are also aware of the inherent limitations implicated in our proposed definitions: First, a significant number of patients does not present within 2 weeks following trauma, making it difficult to detect signs of acute ruptures on ultrasound or MRI. Second, even if the patient presents in time, imaging modalities may not be available in time, especially in rural areas. Third, the exact trauma mechanism is scarcely comprehensible ex post, as the patient may not recall the exact details during the trauma. However, we believe, that it is of critical importance to attain guidelines for the definition of the terms acute and traumatic in the context of rotator cuff tears to be able to compare outcomes of future studies.

## Conclusion

The term “acute” should be reserved for RCT showing muscle edema, wavelike appearance of the central part of the torn tendon, and joint effusion, which typically requires adequate imaging within 2 weeks from trauma. Repair of acute tears should occur within 8 weeks from trauma to benefit from possibly superior biological healing capacities. The term “traumatic” should be used for a sudden onset of symptoms in a previously asymptomatic patient, triggered by an adequate trauma, e.g., a fall on the retroverted arm with an axial cranioventral force or a traumatic shoulder dislocation.

## Data Availability

Not applicable.

## Abbreviations

bFGF: Basic fibroblast growth factor
c: Comparative study (within the MINORS-Score)
IGF-1: Insulin-like growth factor-1
IL-6: Interleukin 6
IL1-ß: Interleukin1-ß
MRI: Magnet resonance imaging
nc: Non comparative study (within the MINORS-Score)
PDGF: Platelet-derived growth factor
RCT: Rotator cuff tear
rct: Randomized controlled trial (within the MINORS-Score)
TGF-β: Transforming growth factor ß
TNF-α: Tumor necrosis factor α
VEGF: Vascular endothelial growth factor
